# Tuberculosis treatment in the private healthcare sector in India: an analysis of recent trends and volumes using drug sales data

**DOI:** 10.1186/s12879-019-4169-y

**Published:** 2019-06-19

**Authors:** Nimalan Arinaminpathy, Deepak Batra, Nilesh Maheshwari, Kishan Swaroop, Lokesh Sharma, Kuldeep Singh Sachdeva, Sunil Khaparde, Raghuram Rao, Devesh Gupta, Bhavin Vadera, Sreenivas A. Nair, Kiran Rade, Sameer Kumta, Puneet Dewan

**Affiliations:** 1 Department of Infectious Disease Epidemiology, Imperial College School of Medicine, London, UK; 2 IQVIA, New Delhi, India; 3 Central TB Division, New Delhi, India; 4 Ministry of Health and Family Welfare, Mumbai, India; 5 Stop TB Partnership, Geneva, Switzerland; 6 WHO India country office, New Delhi, India; 7 Bill and Melinda Gates Foundation, New Delhi, India; 8 Independent consultant, Seattle, USA

**Keywords:** Tuberculosis, Private sector, India

## Abstract

**Background:**

There is a pressing need for systematic approaches for monitoring how much TB treatment is ongoing in the private sector in India: both to cast light on the true scale of the problem, and to help monitor the progress of interventions currently being planned to address this problem.

**Methods:**

We used commercially available data on the sales of rifampicin-containing drugs in the private sector, adjusted for data coverage and indication of use. We examined temporal, statewise trends in volumes (patient-months) of TB treatment from 2013 to 2016. We additionally analysed the proportion of drugs that were sold in combination packaging (designed to simplify TB treatment), or as loose pills.

**Results:**

Drug sales suggest a steady trend of TB treatment dispensed by the private sector, from 18.4 million patient-months (95% CI 17.3–20.5) in 2013 to 16.8 patient-months (95% CI 15.5–19.0) in 2016. Overall, seven of 29 states in India accounted for more than 70% of national-level TB treatment volumes, including Uttar Pradesh, Maharashtra and Bihar. The overwhelming majority of TB treatment was dispensed not as loose pills, but in combination packaging with other TB drugs, accounting for over 96% of private sector TB treatment in 2017.

**Conclusions:**

Our findings suggest consistent levels of TB treatment in the private sector over the past 4 years, while highlighting specific states that should be prioritized for intervention. Drug sales data can be helpful for monitoring a system as large, disorganised and opaque as India’s private sector.

## Background

In India, the country with the world’s largest burden of tuberculosis (TB), the treatment of TB is dominated by the private healthcare sector [1–4]. With a general lack of adherence support, TB patients being managed in this sector face poorer treatment outcomes and an elevated risk of recurrent TB, than those treated in the public sector [5, 6]. In planning interventions to reach these patients - such as the provision of free, high-quality, publicly-funded TB drugs [7] - it is important first to understand the true scale of the problem.

However, the private sector is opaque: it is vast, largely unstructured and highly fragmented [2, 8], and a systemic lack of reporting by private providers means that there is limited information for the true burden of TB being managed in this sector. Moreover, such conditions pose key challenges in gaining objective estimates of the true TB burden in India [9, 10]. In the long term, any sustainable solution will require systematic surveillance and reporting of TB in the private sector, to the same extent as that in the public sector. Recent initiatives have demonstrated effective mechanisms for doing so [11], but it will take time for such approaches to be optimised and scaled up. Until such time there is a pressing need for alternative approaches, to cast light on ongoing TB treatment activity in the private sector.

In this context, recent work showed the potential value of analysing commercially available data on drug sales in the private sector [3, 4]. Earlier analysis showed, for example, how TB drug sales in the private sector in 2014 suggested a higher TB burden in India than had hitherto been recognised [4]. This work examined treatment volumes in single years and so - while illuminating the sheer volume of treatment in the private sector - did not address trends over time. Here we build on this earlier work to capture such trends. Using comprehensive national and state-specific data, we examined the changes in TB treatment volumes from 2013 to 2016. We additionally explored the types of dosage forms typically sold in the private sector, contrasting fixed-dose combinations and co-blistered packs (which are designed to simplify treatment regimens and thus promote adherence [12, 13]) against drugs sold as ‘single salt’, or loose pills. Finally, we analysed the statewise variation in the extent to which the private sector dominates TB treatment, to explore which states may have the highest priority in future interventions to address the private sector in India. This work illustrates how such data could be used to cast light on the spatial and temporal dynamics of private sector TB treatment, in India and elsewhere.

## Methods

We used state-specific data from 2013 to 2016, capturing the sale of all pharmaceutical drugs in the private sector in India during this time, commercially procured from IQVIA™. IQVIA routinely collects commercial data from a recruited panel of stockists, capturing drug sales to retailers, hospitals, dispensing doctors, and other providers. We performed the analysis for all 29 states in India. For ease of exposition we aggregated the smaller, 7 union territories into their geographically closest states. We computed national-level estimates by aggregating over all states.

We identified the ‘volume of TB treatment’ as the number of patient-months of rifampicin-containing products in a given year, that were prescribed for TB. The methodology for estimating TB treatment volumes from drug sales data is described in detail elsewhere [4]. In brief, the drug sales data involves over 190 different rifampicin-containing products. As each product represents different doses, numbers of pills, and other factors, they vary widely in terms of the number of patient days of treatment represented by a single unit. For each product we used data from the IQVIA Medical Health Audit, a large database of prescriptions collected by IQVIA from a panel of private sector providers (approx. 6 k providers) primarily based in urban cities. For this database, approx. 800 k-1mn prescriptions are collected every month. We used this database to determine the associated number of patient days of TB treatment, and to adjust for the proportion of prescriptions that were for TB. Changes in data collection methodology from 2015 onwards have the potential to introduce artefacts in the estimates; to control for these changes, we used only aggregated prescription audit data from 2013 to 2014, applying these to all years under study. Combining this with IQVIA data for units of each product sold, we estimated the total patient-months of TB treatment captured by IQVIA data in a given year. Finally, we adjusted for the proportion of drug sales that are captured by IQVIA data, drawing from IQVIA studies comparing reported data against manufacturer records.

Capturing uncertainty in each of these inputs with probabilistic distributions, we used Monte Carlo simulation to estimate uncertainty in the calculated treatment volumes. To assess any temporal trends, we took advantage of the Monte Carlo simulations to estimate the ratio in drug sales between two given years [14]. We evaluated the 2.5th, 50th and 97.5th percentiles of these proportions to assess statistical evidence for trends over time, denoting these uncertainty estimates as the ‘credible interval’ (where this interval contains 1, there is no evidence at the 5% significance level, for a difference between years). The analysis was performed using Python v 3.6.4.

For comparison with the public sector, we used publicly available, statewise notifications to the Revised National Tuberculosis Control Programme (RNTCP) in 2016 [15]. To enable comparability we converted notifications to estimated patient-months of treatment, assuming each new and previously treated patient to represent 6 and 9 patient-months of treatment, respectively [16]. We explored in which states the private sector has the greatest ‘market share’, i.e. supplying the greatest proportion of overall TB treatment. We also explored which states accounted for the greatest proportion of national-level private sector drug sales. State level population for 2016 was sourced from the 2011 Census of India.

## Results

**Fig. 1:**
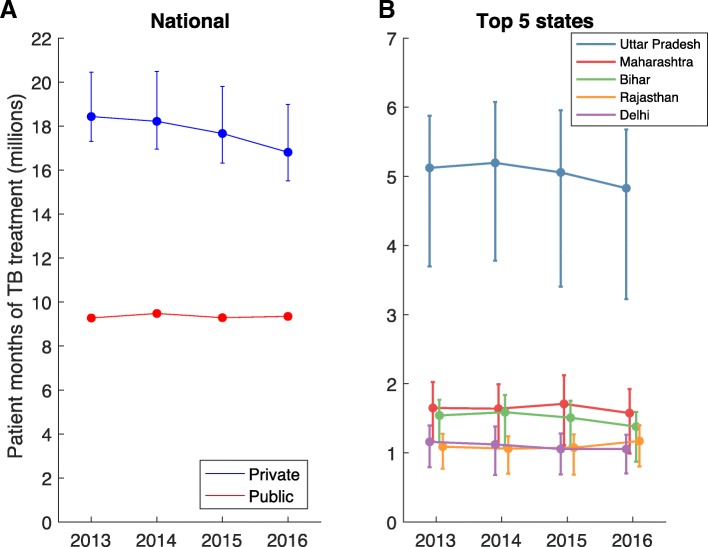
Temporal trends in the volume of TB treatment supplied through the private sector (annual patient-months), since 2013. (**a**) National-level trends. (**b**) Decomposition of national-level trends into the 5 states in India with the greatest volumes of private-sector TB treatment

Figure 1 shows the trends from 2013 to 2016, in the patient-months of TB treatment supplied through the private sector in India. The left-hand panel displays trends on a national level, while the right-hand panel shows a disaggregation by state. On the national level, trends suggest a slight secular decline in TB drug sales by the private sector. Relative to yearly uncertainty, however, this decline does not appear to be significant. The ratio of drug sales in 2016 to 2013 was 0.90 (95% credible intervals 0.81–1.06). Similarly, no significant or meaningful differences emerged in any state 2013–2016, with the top 5 states by drug sales volumes shown in Fig. 1b.

**Fig. 2:**
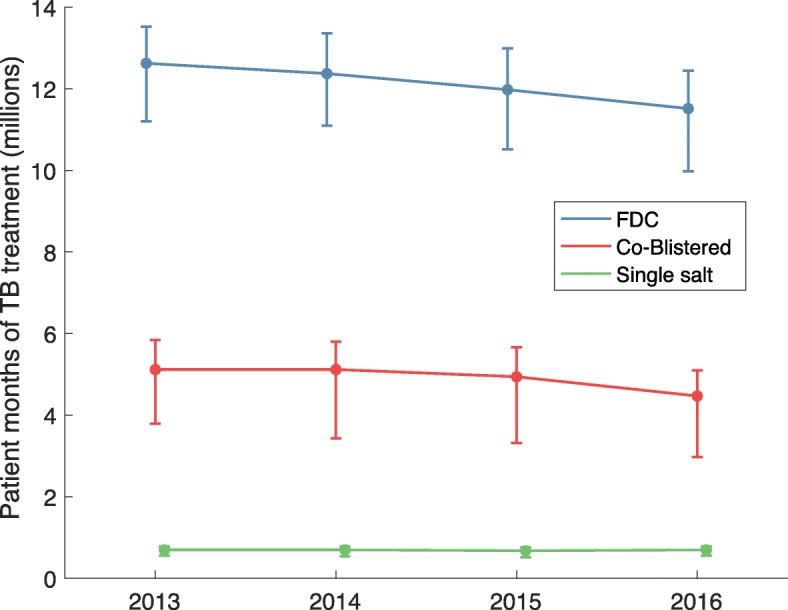
Drug sales by product form. As in Fig.1, these results refer to all rifampicin-containing drugs. ‘FDC’ stands for ‘Fixed Dose Combination’. Both FDCs are co-blistered drugs have the advantage of simplifying TB treatment, compared with single salt (i.e. loose pill) formulations

Figure 2 further disaggregates these trends by dosage form, comparing fixed-dose combinations (FDCs) and co-blistered combinations (CBCs) with loose pills (‘single salt’). The former two are designed to facilitate co-administration of the different drugs involved. The figure suggests that drugs sold in a single salt account only for a small proportion (2–3%) of overall TB treatment volumes, and have remained roughly constant over this period.

**Fig. 3:**
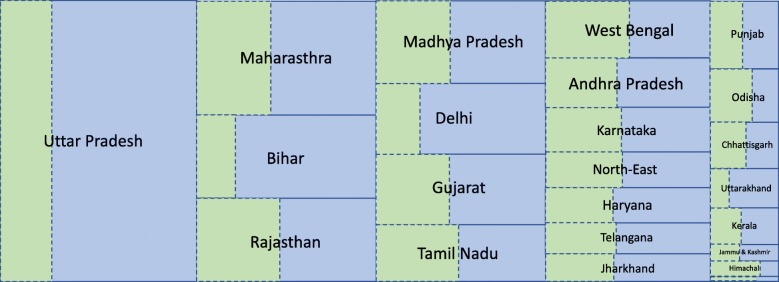
Schematic illustration of patient-volumes of TB treatment in each state. Areas are proportional to total patient-months of treatment in 2016: green segments show public-sector treatment volumes, while blue segments show the private sector. States are listed, from left to right, and top to bottom, in decreasing order of total TB treatment volume (public and private). The state at bottom right is Goa. See caption, Table 1, for how smaller states and union territories are aggregated into these major states

**Fig. 4:**
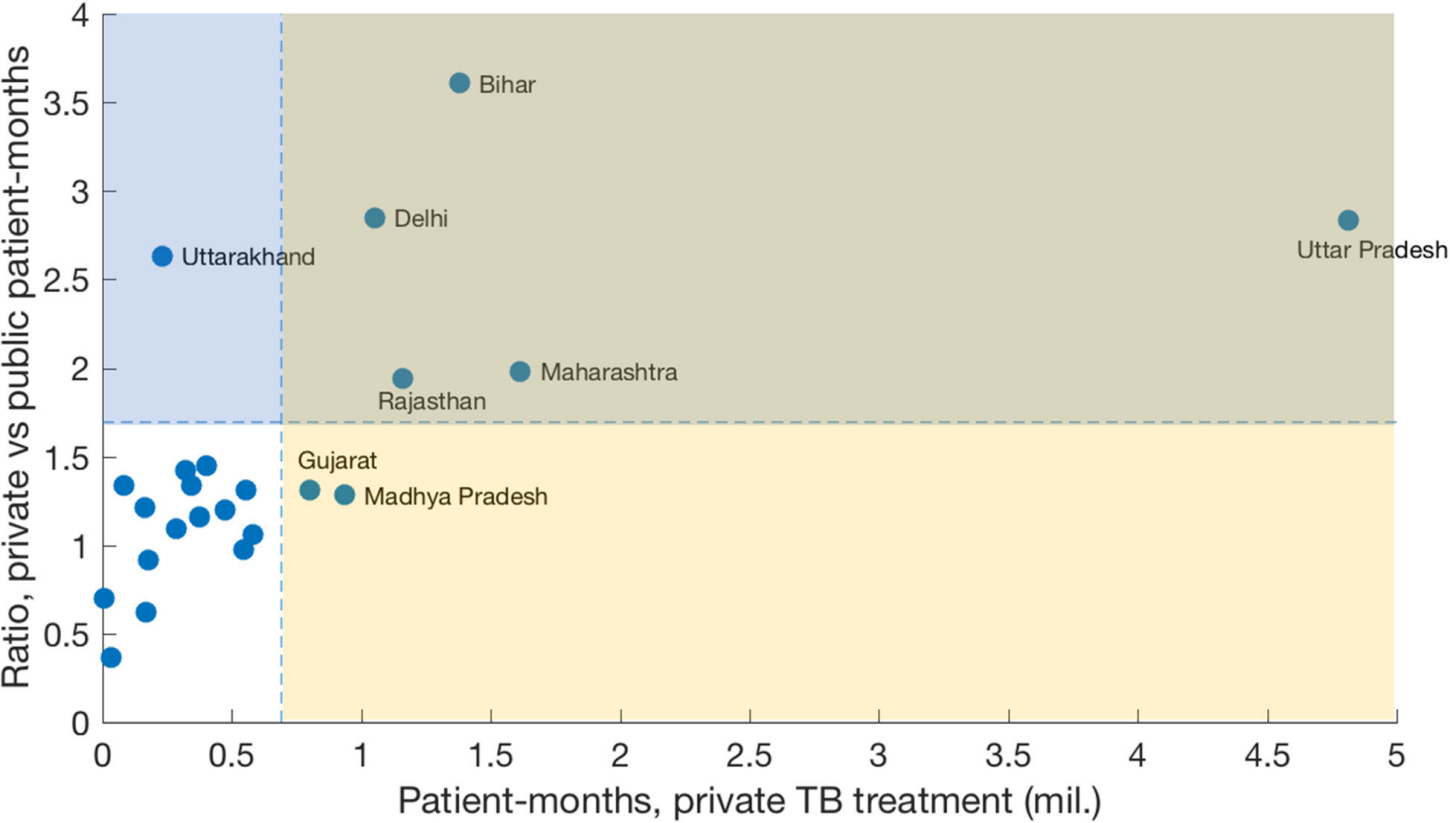
Ordering of states by different measures of priority. Dots show median estimates for each state, omitting uncertainty intervals for clarity. The yellow-shaded region (including the shaded overlap at top right) shows those states that account for over 70% of national-level private sector TB treatment volume. Interventions in these states would have the greatest impact on the size of the private sector nationally. By contrast, the blue-shaded region (including the shaded overlap) shows those states in which the private sector dominates most over the public sector (here, showing the top 6 states for illustration). These states might therefore be seen as having the greatest ‘local need’

Finally, to identify ‘priority’ states for intervention, we conducted a state wise comparison of TB treatment volumes in the public and private sectors in 2016. Table 1 shows quantitative estimates: these are visualised in Fig. 3, which displays areas proportional to volumes of TB treatment in each state. The figure illustrates that Uttar Pradesh, as well as having the greatest share of national-level, private sector treatment (Fig.1b), is also amongst the states where the private sector has the greatest ‘market share’, along with Bihar and Delhi. Figure 4 presents another visualisation, showing two measures by which states may be prioritised for intervention: whether by their contribution to national-level private sector treatment volumes (x-axis), or by the extent to which the private sector within the state dominates over the public sector (y-axis). The figure illustrates that, by both measures of priority, five states (Bihar, Delhi, Rajasthan, Maharashtra, Uttar Pradesh) would call for the highest priority in interventions to address the private sector. These ‘priority states’ are not necessarily the most populous: for example, West Bengal has a greater population than Rajasthan and Delhi, and yet falls in the lower-left (unshaded) quadrant of Fig. 4. Table 1 gives the quantitative estimates behind Figs. 3, 4.

**Table 1:** State-wise comparison of RNTCP and private-sector TB drug supplies. Here, ‘market share’ denotes the proportion of total patient-months of TB treatment (RNTCP along with private) in a given state

State	RNTCP patient-months (mil.)	Private patient-months (mil.)	Private patient-months (per 100 k state population)	RNTCP market share (%)	Private market share (%)	Private/RNTCP ratio
Andhra Pradesh	0.42	0.56 (0.41, 0.76)	651.0 (502.0, 876.0)	43.0 (36.0, 51.0)	57.0 (49.0, 64.0)	1.3 (0.97, 1.8)
Bihar	0.38	**1.4 (1.2, 1.8)**	1320.0 (1110.0, 1740.0)	22.0 (17.0, 25.0)	**78.0 (75.0, 83.0)**	**3.6 (3.0, 4.8)**
Chhattisgarh	0.2	0.18 (0.14, 0.24)	703.0 (550.0, 978.0)	52.0 (45.0, 58.0)	48.0 (42.0, 55.0)	0.91 (0.73, 1.2)
Delhi	0.37	1.0 (0.83, 1.4)	**6300.0 (4950.0, 8190.0)**	26.0 (21.0, 31.0)	**74.0 (69.0, 79.0)**	**2.8 (2.2, 3.7)**
Goa	0.01	0.0072 (0.005, 0.011)	494.0 (342.0, 797.0)	**59.0 (47.0, 67.0)**	41.0 (33.0, 53.0)	0.71 (0.49, 1.1)
Gujarat	0.61	0.8 (0.64, 1.1)	1320.0 (1050.0, 1800.0)	43.0 (36.0, 49.0)	57.0 (51.0, 64.0)	1.3 (1.0, 1.8)
Haryana	0.28	0.4 (0.32, 0.53)	1600.0 (1280.0, 2150.0)	41.0 (34.0, 46.0)	59.0 (54.0, 66.0)	1.5 (1.2, 1.9)
Himachal Pradesh	0.093	0.034 (0.025, 0.054)	506.0 (364.0, 862.0)	**73.0 (63.0, 79.0)**	27.0 (21.0, 37.0)	0.37 (0.27, 0.58)
Jammu and Kashmir	0.061	0.082 (0.063, 0.13)	644.0 (476.0, 992.0)	43.0 (32.0, 50.0)	57.0 (50.0, 68.0)	1.3 (1.0, 2.1)
Jharkhand	0.23	0.33 (0.26, 0.46)	971.0 (798.0, 1330.0)	41.0 (33.0, 46.0)	59.0 (54.0, 67.0)	1.4 (1.2, 2.0)
Karnataka	0.4	0.48 (0.35, 0.65)	769.0 (557.0, 1060.0)	45.0 (38.0, 53.0)	55.0 (47.0, 62.0)	1.2 (0.87, 1.7)
Kerala	0.13	0.16 (0.12, 0.23)	473.0 (355.0, 673.0)	46.0 (37.0, 54.0)	54.0 (46.0, 63.0)	1.2 (0.86, 1.7)
Madhya Pradesh	0.73	0.93 (0.75, 1.3)	1290.0 (1050.0, 1770.0)	44.0 (36.0, 49.0)	56.0 (51.0, 64.0)	1.3 (1.0, 1.8)
Maharashtra	0.81	**1.6 (1.3, 2.1)**	1440.0 (1100.0, 1960.0)	34.0 (28.0, 39.0)	66.0 (61.0, 72.0)	1.9 (1.5, 2.6)
North East	0.32	0.37 (0.29, 0.55)	878.0 (686.0, 1180.0)	46.0 (37.0, 52.0)	54.0 (48.0, 63.0)	1.2 (0.92, 1.7)
Odisha	0.27	0.17 (0.13, 0.24)	397.0 (307.0, 582.0)	**61.0 (53.0, 67.0)**	39.0 (33.0, 47.0)	0.63 (0.49, 0.9)
Punjab	0.26	0.29 (0.24, 0.38)	991.0 (812.0, 1310.0)	48.0 (41.0, 53.0)	52.0 (47.0, 59.0)	1.1 (0.9, 1.4)
Rajasthan	0.6	1.2 (0.95, 1.5)	1710.0 (1360.0, 2290.0)	34.0 (28.0, 39.0)	66.0 (61.0, 72.0)	1.9 (1.6, 2.6)
Tamilnadu	0.55	0.59 (0.43, 0.76)	790.0 (596.0, 1070.0)	48.0 (42.0, 56.0)	52.0 (44.0, 58.0)	1.1 (0.78, 1.4)
Telangana	0.26	0.34 (0.26, 0.47)	980.0 (725.0, 1370.0)	43.0 (35.0, 50.0)	57.0 (50.0, 65.0)	1.3 (0.99, 1.8)
Uttar Pradesh	1.7	**4.8 (4.0, 6.3)**	**2400.0 (1980.0, 3310.0)**	26.0 (21.0, 30.0)	**74.0 (70.0, 79.0)**	**2.9 (2.4, 3.7)**
Uttarakhand	0.088	0.23 (0.19, 0.33)	**2290.0 (1850.0, 3130.0)**	27.0 (21.0, 31.0)	73.0 (69.0, 79.0)	2.6 (2.2, 3.7)
West Bengal	0.56	0.55 (0.43, 0.77)	606.0 (470.0, 838.0)	51.0 (42.0, 57.0)	49.0 (43.0, 58.0)	0.98 (0.76, 1.4)
National	9.3	17.0 (16.0, 19.0)	1350.0 (1250.0, 1540.0)	36.0 (33.0, 38.0)	64.0 (62.0, 67.0)	1.8 (1.7, 2.0)

Numbers in bold indicate the three most important states, judged by their median estimates, in a given column. Smaller states and union territories are aggregated as follows: Chandigarh (aggregated with Punjab), Dadra and Nagar Haveli (with Gujarat), Daman and Diu (with Gujarat), Lakshadweep (with Kerala), and Puducherry and Andaman & Nicobar Islands (with Tamil Nadu)

## Discussion

For an objective measure for the performance of future interventions, as well as an understanding of the burden of TB being managed by the private sector, there is a pressing need to systematically monitor TB treatment in this sector. Here we aimed to address this challenge, using comprehensive data on TB drug sales in the private sector in India. Our work builds on previous analysis of private sector drug sales [3, 4], including a seminal study combining data from Pakistan, the Philippines, India and Indonesia [3]. To our knowledge, the present study is the first to assess trends over time, in a given setting.

Our results suggest that in India there may have been a minimal decline in recent years in TB treatment volumes in the private sector, with interannual variation remaining within the bounds of intra-annual uncertainty (Fig.1). This highlights the urgency of addressing the private sector, and the inadequacy of pilot efforts to affect the national challenge. Nonetheless, it is worth observing that these trends are taking place against a backdrop of significant efforts in India’s TB response to the private sector. Notably, the last three years have seen a marked escalation in the notification of TB patients from the private sector, arising largely from a nationwide push to capture private notifications; strong state-level efforts in 3 states (Maharashtra, Gujarat, and Kerala); and particularly potent new approaches for engaging with the private sector that have emerged from pilot programs [15, 17]. Furthermore, the recently-published National Strategic Plan has laid out a far-reaching vision for the future of India’s TB response, with private sector engagement at its core [18]. Tracking drug sales data may be especially crucial to monitor the success of these efforts, as the approach being taken by India is to displace private drug sales with publicly-provisioned TB treatment distributed through private providers and chemists. This would be expected, if successful, to affect large volumes of private TB treatment. Progress could be monitored regularly state-wise by the combination of rising TB notifications and falling private TB treatment.

Our results also cast light on the types of drugs typically prescribed for TB treatment in the private sector: despite concerns about the overall quality of TB care in the private sector [1, 19, 20], our results suggest that prescription practices for TB are showing encouraging signs, with private providers overwhelmingly prescribing combination products, i.e. FDCs and CBCs. These dosage forms are valuable in minimizing prescription errors, simplifying TB treatment regimens, and facilitating adherence [12, 13]. However, with first-line regimens typically 6 months in duration, the lack of adherence support and monitoring for privately-treated patients creates total uncertainty and great concern for treatment completion among patients treated in the private sector. Such concerns could be addressed by extending adherence monitoring to privately-notified TB patients, and are facilitated by the emergence of new, low-cost adherence support mechanisms. Novel adherence tools utilise the blister packaging for FDCs and CBCs to facilitate a patient’s daily contact with a call-centre-based adherence tracking system, and are in widespread use in India [21]. This is another aspect in which the current dominance of blister-packed FDCs in the private TB drug market appears to be an encouraging sign, for the future implementation of these and other adherence support mechanisms.

Our analysis also illustrates important statewise variation (Fig.3, Table 1), with just 7 states (Uttar Pradesh, Maharashtra, Bihar, Rajasthan, Madhya Pradesh, Delhi, and Gujarat) accounting for over 70% of private sector drug sales in India. These findings also highlight different approaches for prioritising states. For example, in Fig. 4, is it better to prioritise those ‘high-volume’ states (listed above) in which addressing the private sector would have the greatest impact on the national-level private market, and largest potential impact on the epidemic? Or is it preferable to address first those smaller populations with the greatest ‘local need’, where the private sector is most dominant over the public (e.g. Uttarakhand)? Any future strategy is likely to involve a combination of these strategies: our findings highlight the potential value of systematic quantification of the burden of TB treated in the private sector, for informing such planning.

We also note again an important limitation of this approach: that true TB burden is measured in terms of numbers of patients, not treatment volumes. This limitation has been extensively discussed previously [4]. There is considerable uncertainty in translating the latter to the former, and so the approach illustrated here should not be interpreted as a substitute for routine surveillance, and for disease burden surveys. India’s forthcoming national prevalence survey will provide invaluable, direct measurement of the true burden of TB in India, as will improved surveillance in the private sector. By comparison, our approach in the present study offers a complementary approach: concentrating instead on ‘market share’ of the TB drug market, this is particularly relevant for interventions involving TB treatment in the private sector.

## Conclusions

It is widely recognised that there will remain major challenges for TB control in India as long as TB treatment is dominated by such a large and fragmented private sector [5]. Together with currently available surveillance tools, approaches such as those presented here could contribute to a comprehensive picture of state of the private sector; how it changes over time; and where interventions are needed most. Such monitoring will be invaluable in future interventions to harness the private sector, for the benefit of TB patients in India and elsewhere.

## Data Availability

The datasets generated during and/or analysed during the current study are available from the corresponding author on reasonable request.

## Abbreviations

CBC: Co-blistered combination
FDC: Fixed-dose combination
RNTCP: Revised National Tuberculosis Control Programme
TB: Tuberculosis
